# Mitral valve restoration using the No-React(R) MitroFix™: a novel concept

**DOI:** 10.1186/1749-8090-7-82

**Published:** 2012-09-04

**Authors:** Frank Oertel, Karl Golczyk, Sigrun Pantele, Vladimir Danov, Manuel Galiñanes, Michael Beyer

**Affiliations:** 1 Heartcentre Augsburg, Augsburg, Germany; 2 Department of Cardiac Surgery, St. Anna University Hospital, Sofia, Bulgaria; 3 Department of Cardiac Surgery, University Hospital Vall d’Hebron, University Autonoma of Barcelona, Barcelona, Spain

## Abstract

**Background:**

Mitral Valve Repair (MVRP) has been shown to be significantly superior to Mitral Valve Replacement (MVR). Since the majority of repairs involve the Posterior Mitral Leaflet (PML) and not the Anterior Mitral Leaflet (AML), the monocuspidalisation of the Mitral Valve (MV) can be achieved with a bio-posterior leaflet that imitates a closed PML. This approach may have the benefit of restoring the competence of the MV without reducing its effective orifice area.

**Methods:**

We have used a new concept and device, the MitroFixTM, to correct MV regurgitation due to pathology of the PML. The device comes with functional sizers both of which have identical shape and size. This allows the surgeon to pre-test the success of the restoration. From December 2006 to October 2011, 51 MitroFixTM devices were implanted at three institutions.

**Results:**

The mean age of the patients (32 males and 19 females) was 67.7 years. 37 of them were in NYHA class III or IV and all patients suffered from severe mitral valve regurgitation (MR). 31 patients underwent combined surgery. Successful implantation of the MitroFix™ device was performed in 51/53 patients.Mean cross-clamp time was 63.6 min (range: 29-118 min). Six patients had additional reconstructive procedures of the AML (chordae transfer, neo-chordae, triangular resection). At discharge, 33 patients showed no MR in the TTE and 17 patients exhibited trivial (I) or moderate (II) MR. The mean gradient was 4.0 mmHg and mean EOA was 2.52cm^2 (range: 1.5-4.0cm2). All patients were classified as being in NYHA class I or II.

**Conclusion:**

The MitroFixTM Mitral Valve Restoration Device is a new concept that offers an effective treatment of MR. The restoration of the mitral valve with the MitroFix™ device offers the advantage of preserving the AML and providing good coaptation with a prosthetic PML. Importantly, this preliminary evaluation indicates a mean effective orifice area ( EOA ) of 2.5cm2 in MV receiving a MitroFix™ device, witch is higher than EOA resulting from MVR or MVRP. The present study has also shown that severe regurgitation due to ischemic/rheumatic MR, endocarditis and complex prolapse of the PML are clear candidates for correction with the MitroFix™. Larger studies and a longer follow up period are needed to validate these promising results.

## Background

Mitral valve repair (MVRP) is the preferred treatment for patients with mitral valve regurgitation (MR) with important advantages over mitral valve replacement (MVR), including: (I) reduced operative mortality, (II) improved long-term survival, (III) better preservation of left ventricular function and hemodynamics, and (IV) greater freedom from endocarditis, thromboembolism, and anticoagulant- related hemorrhages [1].

Despite these well-established benefits, however, less than 65% of diseased mitral valves are repaired [2], largely owed to the many surgical/technical challenges associated to this procedure, and also to a variety of clinical scenarios which make the patient ill-suited for conventional repair [3]. The rate of repair could vary from 10% to 90% depending on the type of mitral valve pathology [4]. Replacement is a very quick and reproducible procedure with predictable result, whereas repair is time consuming, requires a considerable experience of the surgeon and it is accompanied by a learning curve.

Even among skilled surgeons, the feasibility and the outcome of MVRP can be highly variable, mainly due to the cause of the dysfunction [4]. Reoperation rates of between 2% and 5% and even higher have been reported at 11 years follow-up [5,6], while the rate of failures during surgery and during the early postoperative period remains unknown.

### Shortcomings of MV annuloplasty

One of the basic techniques of MVRP is the use of an annuloplasty ring to stop or reverse annular dilatation [7]. The annuloplasty ring increases coaptation by decreasing the anterior-posterior dimension of the mitral annulus. This transformed the MV into a single leaflet mechanism (monocuspidalisation) with a frozen posterior leaflet (PML) serving as a buttress for the closing even in non-diseased valves [8]. The result of this approach is a reduction of the EOA.

### Potential benefit and conditions that could be suitable for the restoration of the MV competence with the MitroFix^TM^ device

The feasibility of MVRP depends on the pathology of the regurgitation. In general more than 90% of the MV [4] with degenerative disease and isolated prolapse of the posterior leaflet (Type II) can be repaired using conventional techniques with good long-term results in terms of freedom from reoperation, bleeding or thromboembolic events [9]. Similar results can be seen in Type I MV regurgitation depending on the underlying disease [10].

By contrast, in patients with a history of rheumatic fever, because of the underlying complex valvular and subvalvular leasions [11], conventional valve repair can be very difficult and the durability of the repair is limited [12]. The possibility of repair depends on the surface area, the pliability and mobility of the anterior leaflet (AML) and the ability of the AML to coapt against the PML. Various complex techniques like commisurotomy, augmentation, decalcification and chordae splitting are used. But because of the progressive disease of the valve and annulus, especially in young patient, the results are rather poor. In old patients the long-term results are better, but repair is still a challenging procedure [4]. The MitroFix^TM^ device may be a potential alternative to restore the competence of the MV with the added benefit of preserving the EOA of the valve.

Results of standard repair in patients with ischemic MR tend to be still worse than in degenerative disease [13] and additional repair options, with the possibility of restoring mitral valve function, are needed [14]. Type IIIb MR is characterized by restricted systolic leaflet motion with preserved leaflet pliability. The basic mechanism of this functional MR is tethering as a result of segmental or global LV dilatation caused by ischemic or non-ischemic dilated cardiomyopathy. Ischemic MR results from restriction of the posterior leaflet motion making it unavailable for coaptation with the anterior leaflet. The MR is caused by changes in the geometry of the LV and MV- apparatus in the absence of structural damage to the valve [15]. Especially, ischemic MR is often caused by asymmetrical tethering, resulting from segmental dilatation following infarction or ischemic dysfunction of the posteromedial papillary muscle. The prevailing consequence is tethering of the PML (P2, P3, PC) and restricted leaflet motion [13]. The surgical approach to attain competence of ischemic MR is to increase or restore leaflet coaptation by myocardial revascularization [13] to prevent further dilatation and remodeling of the LV associated to restrictive MV annuloplasty. The annuloplasty is performed by downsizing 1 or 2 sizes to force the AML to coapt against the restricted PML. But downsizing does not relieve tethering – it is just shifting the posterior annulus anterior to achieve coaptation [15].

Despite good long-term survival for patients undergoing MVRP and high freedom from reoperation, many patients with ischemic MR experience a deterioration of the regurgitation during the first six months following the procedure. McGee et al. [16] reported that in patients with ischemic MR undergoing repair, the proportion of those with 0 or 1+ mitral regurgitation decreased from 71% to 41% during the first six months following surgery, whereas the proportion of those with 3+ or 4+ regurgitation increased from 13% to 28%. Similar recurrence rates of severe MR after primal successful repair are reported by other authors [15,17,18]. Restrictive annuloplasty is also accompanied by the risk of functional MV stenosis [19]. These studies suggest that conventional repair of ischemic MR may be suboptimal. Hence, many surgeons still prefer MVR in complex rheumatic, degenerative or ischemic MR, with the view that “*good replacement is better than bad repair*” [20]. Therefore, any innovation (device or a technique), that makes MVRP easier and feasible and also more accessible to the majority of surgeons is very welcome.

The intention of our study is to evaluate whether the MitroFix™ Device leads to comparable good results in elective cases as MVR. The design of the device was motivated by the following key observations:

### Principles for the design of the MitroFix™ device

The design of the device was motivated by the following key observations:

1. Repair of the mitral valve is always preferable to replacement

2. The anterior leaflet contributes 70% of the mitral valve EOA and, hence, patients who have a normal anterior leaflet, the AML should be preserved

3. New posterior leaflet mimicking normal posterior leaflet in closed position can simplify mitral valve restoration while achieving EOA higher than mitral valve replacement or repair

4. The optimal repair system should be simple making the procedure more reliable and accessible to the majority of surgeons

## Methods

### Description of the MitroFix™

**Figure 1:**
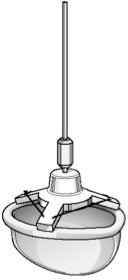
The Mitrofix™ device.

**Figure 2:**
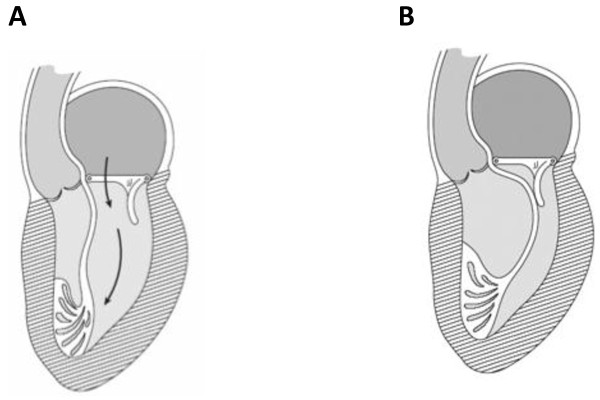
The MitroFix™ device in open (A) and coapting (B) position with the anterior leaflet of the MV.

The MitroFix™ consists of a ”D” shaped device with a curved surface made of medical grade polymer covered with pericardium (Figure 1). This shape is designed to mimic the posterior leaflet in the closed position, forming a “buttress” against which the anterior leaflet can coapt (Figure 2). The device is available in 28, 30, 32, 34, 36 and 38 mm sizes.

### Description of the MitroFix™ sizer

**Figure 3:**
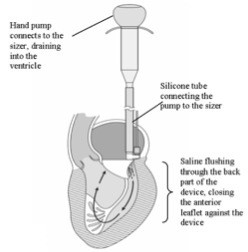
Schematic view of the Mitrofix™ sizer.

**Figure 4:**
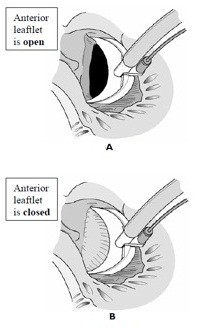
MitroFix™ sizer (view from above) placed with the anterior leaflet of the mitral valve in open (4A) and closed (4B) position.

The Mitrofix™ sizer (Figures 3 &4A and B) is an integral part of the Mitrofix™ repair system. The functional sizer both enables the surgeon to accurately measure the size of the annulus, and exactly mimics the Mitrofix™¢ thereby allowing the surgeon to test the function and integrity of the repair after inserting two commissural sutures and pledgeted sutures of the posterior ring. Proper device size selection is an important part of repair using the Mitrofix™ system. A silicon tube is attached to the luer on the atrial side of the D-shaped sizer. Saline is injected through the luer and exits at the ventricular side. The ventricle fills and is pressurized to test the repair prior to implantation. This gives an important insight onto the feasibility of the repair with the Mitrofix™ device.

From December 2006 to October 2011, 51 Mitrofix™ devices were implanted at three different centers (Augsburg/Germany n = 34, Sofia/Bulgaria n = 12, Barcelona/Spain n = 5). The patients’ characteristics are shown in Table 1. The mean age of the patients (30 males and 21 females) was 67,72 years (range 52-85 years). Of them, 37 were in class III or IV of the NYHA and all patients suffered from severe MR. 31 patients had combined surgery. Successful implantation of the MitroFix™ device was performed in 51/53 (96,22%) patients. The indications for the use of the Mitrofix™ device are shown in Table 2 and were: (I) severe destruction of the PML by endocarditis or degenerative disease, (II) rudimentary or calcified PML, and (III) restrictive ischemic or rheumatic PML.

**Table 1:** Patient’s characteristics - 3 Centers - (MitroFix© Device)

**N valid**			
Age at surgery (mean)		51	67,58
			Range:52-
Gender		51	
	Males		32 (62,7%)
	Females		19 (37,3%)
Preoperative clinical status			
NYHA class		51	
	II		6 (11,8%)
	II-III		7 (13,7%)
	III		24 (47,1%)
	III-IV		6 (11,8%)
	IV		8 (15,7%)
Atrial fibrillation		51	21 (41,2%)
Additional cardiac diseases		51	
	None		20 (39,2%)
	Cardiac artery disease		18 (35,3%)
	Aortic valve disease		4 (7,8%)
	Tricuspid valve disease		13 (25,5%)
	Persistant foramen ovale		3 (5,9%)
	Redo		2 (3,9%)
	AAA		1 (2,0%)
Mitral regurgitation (preop)			
	III		17 (33,3%)
	III-IV		10 (19,6%)
	IV		24 (47,1%)

**Table 2:** Surgical data (MitroFix© Device)

**N valid**			
Mitral pathology		51	
	calcificated		3 (5,9%)
	endocarditis		4 (7,8%)
	ischemic		16 (31,4%)
	myxomatous		3 (5,9%)
	fibrotic		1 (2,0%)
	rheumatic,+1 endocarditis		16 (31,4%)
	degenerative		8 (15,7%)
PML Type (Carpentier classification)		51	
	I		2 (3,9%)
	II		14 (275)
	(+ Endocarditis		5 (9,8%)
	IIIa		14 (27,5%)
	IIIb		16 (31,4%)
AML structural alteration		51	
	none		39 (76,5%)
	Type II		7 (13,7%)
	Fibrosis		5 (9,8%)

### Surgical implantation technique

All operations were performed through a median sternotomy using standard cardiopulmonary bypass and patients were monitored with TEE. After exposure of the MV and analysis of the underlying pathology the anterolateral and the posteromedial trigones of the valve were identified and one stitch in each of these areas was applied. The size of the AML was measured by using a Carpentier Physio Ring Sizer. Then the corresponding MitroFix-sizer was positioned at the posterior annulus and the competence of the valve was assessed by injecting saline through the sizer and into the ventricle. Prolapse of the anterior leaflet did not exclude the use of the Mitrofix™ device, but the abnormality may required separate correction of the anterior leaflet pathology. Thus the presence of prolapse of the anterior leaflet may require transposition of chordae, implantation of neo-chordae, resection or other surgical techniques.

**Figure 5:**
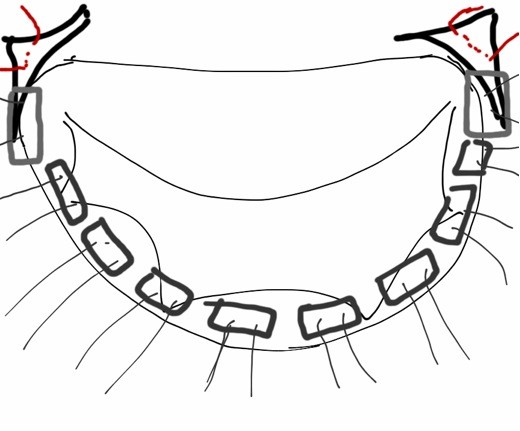
Surgical technique showing the insertion of plegeted sutures through the posterior annulus, then passed through the ring of the MitroFix™ and lowered into position.

Once the size has been chosen, mattress sutures with pledget are threaded from the ventricular side through the leaflet to annulus. Attaching the posterior leaflet to the annulus preserves the subvalvular apparatus and reinforces the posterior annulus. While threading the sutures as seen in Figure 5, there is no need to perform an annuloplasty and undersizing (more than one size is not recommended). The other arm of the sutures is threaded through the middle part of the annulus of the device. Special care was taken at both commissures, not to compromise the commissural chordae. The MitroFix device is then implanted like a prosthetic valve. The anterior bridge, which is very flexible, was cut in between the stitches placed in the area of the trigones mimicking an open ring; although, alternatively, the anterior bridge of the device can be left intact and fixed to the anterior annulus. It is worth noting that the two trigonal sutures are important to add additional strength and stability to the ring.

## Results

Table 3 shows that the MitroFix device was successfully implanted in 51 of the 53 operated patients (96,2%). Of the 2 intraoperative failures, one patient received a biological valve and another one underwent a complex mitral valve repair. The MitroFix device sizes most used were the 32 mm and 34 mm. Almost 60% of the patients received one or more associated cardiac surgical procedure. The mean cardiopulmonary and aortic cross clamp times are also shown in Table 3. Only one patient with endocarditis died >30 days postoperatively of septic shock.

**Table 3:** Intraoperative Data

**Device size**	**51**		
	28		1 (2,0%)
	30		8 (15,7%)
	32		14 (27,5%)
	34		18 (35,3%)
	36		10 (19,6%)
Additional procedure		51	
	none		20 (39,2%)
	CABG		14 (27,5%)
	AVR		3 (5,9%)
	TVR		9 (17,6%)
	RFA		6 (11,8%)
	other		9 (17,6%)
CPB time (min) (mean/Range)		51	92,8 (47-167)
Aortic XC time (min) (mean/Range)		51	64,5 (29-118)
Failed reconstuctio/switch		53	2 (3,8%)

As shown in Table 4, the early intraoperative and postoperative TEE demonstrated none or only trivial residual MR in 90,2% of the patients. In 5 patients more than trivial MR was detected. Importantly, the mean EOA measured by PHT was 2,51 cm2, with a peak gradient of 8,28 mmHg and a mean gradient of 4,0 mmHg during the first postoperative control before discharge.

**Table 4:** Mitral valve function after surgical correction as assesed by intraoperative and/ or postoperative TEE

**Mitral regurgitation**	**51**		
	0		33 (64,7%)
	0-I		11 (21,6%)
	I		2 (3,9%)
	I-II		2 (3,9%)
	II		2 (3,9%)
	III		1 (2,0%)

Postoperative result.

Three patients (5,9%) had intraoperative / postoperative mitral regurgitation II or III. Patient 1 have had a residual MR II and died postoperatively due to multiorganic failure caused by multimorbidity and the fact that he was operated in a myocardial infarction situation.

The second patient from the Sofia-group had a residual MR II. It was an operation for a myxomatic valve, the patient died due to multiorganic failure after replacement of the MitroFix by a mechanical valve. Since then we postulated a myxomatic valve to be a contraindication for it can cause a systolic anterior motion, SAM.

The third patient coming from the Spanish group have had a MR III with severe endocarditic. He died 37 days postoperatively due to multiorganic failure caused by septic prostration.

There was no thrombosis; the anterior leaflet is still left. The anticoagulation is comparable as after MVR: 3 month cumarine (Marcumar) aiming INR 2,5, then there is no further anticoagulation required if the patient shows sinus rhythm, eventually ASS 100 mg per day.

## Discussion

The present study has demonstrated that severe mitral valve regurgitation mainly due to diseased posterior leaflet can be repaired successfully using the MitroFix™device. The device replaces the diseased posterior leaflet allowing the preservation of the anterior leaflet providing excellent early functional and clinical results. This is a new concept in the surgical treatment of the mitral valve that allows restoration of the valve competence whilst maintaining the EOA and preserving the anterior leaflet and the subvalvular apparatus. The importance and clinical relevance of this new concept in corrective mitral valve surgery warrants further discussion

Restoration of mitral valve competence by surgical repair in the presence of a severely dysfunctional or destroyed PML can be challenging even in the hands of cardiac surgeons highly experienced in mitral valve repair surgery. Furthermore, reparative surgery of the severely altered PML does not prevent the progression of the disease and the recurrence of MR. In this context, the use of the MitroFix™ device, which transforms the MV into a monocuspid valve like it is done in the classic reconstruction [8], can be a better alternative to valve replacement, thus avoiding the complications associated with prosthetic valve replacement (e.g. anticoagulation, thrombosis, degeneration). It is also likely that, in contrast with reconstruction of severely altered PML, the use of the MitroFix™ would result in fewer reoccurrence of MR and the need for redo surgery. As demonstrated in this study, the MitroFix™ may be particularly useful in the presence of rheumatic or ischemic MR, where owing to the progression of the disease the middle- and long-term results of reconstructive MV are unsatisfying [6,15,21]. Similarly, the MitroFix™ may also be useful for the correction of degenerative MV disease that often requires complex valve reconstruction and which results are less predictable [9]. Certainly, we showed that in more than 90% of the cases a competent MV could be restored, especially in restrictive type III MR, whereas other investigators have reported 15-30% of residual MR immediately after valve repair with annuloplasty [15].

The specific design and the implantation technique enable the surgeon to implant a rather large MitroFix™ -device without the necessity of performing a restrictive annuloplasty, thus preserving the EOA of the MV. In the absence of pathologies of the AML, the whole leaflet serves as the opening surface of the valve; although in the presence of additional changes on the AML (prolapsuis, destruction), parts of the PML (chordae, segments) can be used to repair the AML. In the later case there is no need to restore the PML that can be completely replaced by the MitroFix™ -device. The use of the MitroFix™ device also results in a shortening of the ischemic and cardiopulmonary bypass times which may be an important factor in complex reconstructions and in the presence of reduced cardiac function. Based on our experience, we see severe type III alteration of the PML as the main indication for the use of the MitroFix™, which from a technical point of view still remains the most challenging MV pathology to be corrected and it will represent the most frequent affectation of the MV in the future [22]. The MitroFix™ may be particularly useful in ischemic MR, where even specially developed annuloplasty rings have not solved the problem of recurrent MR caused by tethering [13] and extreme downsizing resulting in substantial reduction of the EOA [23]. Both problems can be overcome by the use of the MitroFix™ device.

## Conclusions

In summary, we have demonstrated that the MitroFix™is a device that can be used in conditions where the posterior leaflet is partially or completely dysfunctional or even destroyed by various pathologies including endocarditis. This is a new concept in the surgical treatment of the mitral valve that allows restoration of the valve competence with maintenance of the EOA and preservation of the subvalvular apparatus and the anterior leaflet. Importantly, the device is also useful to restore competence of the ischemic mitral valve where the tethering of the leaflets makes difficult the repair by conventional surgical approaches.The use of the MitroFix™has the additional advantages of shortening of the cross-clamp time that is deemed important for the mortality and morbidity of cardiac patients [24]and, by being an easy and rapid learning of the technique, facilitating its use by a larger number of surgeons with less experience in reparative mitral valve surgery.

However, it should be clarified that in the present study a small number of patients followed-up for a short period of time were included and that there is a need for a larger number of treated patients and follow-up for longer periods of time to evaluate the value of the device.

## Competing interests

The authors declare that they have no competing interests.

## Authors’ contributions

FO performed the surgery, designed the study, analysed the results and participated in writing the manuscrupt. KG analysed the results, carried out the statistical analyses and participated in writing the manuscrupt. SP contributed to the collection of data. VD and MG performed the surgery. MB supervised the project. All authors read and approved the final manuscript.
